# Extracellular vesicles as potential biomarkers and therapeutic approaches in autoimmune diseases

**DOI:** 10.1186/s12967-020-02609-0

**Published:** 2020-11-12

**Authors:** Kaiyuan Xu, Qin Liu, Kaihui Wu, Liu Liu, Maomao Zhao, Hui Yang, Xiang Wang, Wenmei Wang

**Affiliations:** Department of Oral Medicine, Nanjing Stomatological Hospital, Medical School of Nanjing University, 30 Zhongyang Road, Nanjing, 210008 China

**Keywords:** Autoimmunity, Biomarker, Therapy, Extracellular vesicle, Exosomes, MicroRNA

## Abstract

Extracellular vesicles are heterogeneous populations of naturally occurring secreted small vesicles. EVs function as signaling platforms to facilitate intracellular communication, which indicates the physiological or pathophysiological conditions of cells or tissues. Considering that EVs can be isolated from most body fluids and that molecular constituents could be reprogrammed according to the physiological status of the secreting cells, EVs are regarded as novel diagnostic and prognostic biomarkers for many diseases. The ability to protect encapsulated molecules from degradation in body fluids suggests the potential of EVs as biological medicines or drug delivery systems. This article focuses on the EV-associated biomarkers and therapeutic approaches in autoimmune diseases.

## Introduction

Extracellular vesicles (EVs) [1], membrane-encapsulated vesicles released by cells, are characterized by lipid bilayer membranes. EVs contain specific biomolecules, including proteins, microRNAs, mRNAs, long noncoding RNAs, cytokines, growth factors, and bioactive lipids [2]. Some of these biomolecules indicate the vesicle origin, and others involve in targeting cells. According to the biogenesis, morphology and dimensions, EVs are classified into (i) exosomes (30–150 nm); (ii) microparticles (MPs; 100–1000 nm); and (iii) apoptotic bodies (1000–5000 nm) [3]. EVs are released by almost all cell types and present in virtually all body fluids, such as blood, urine, milk, saliva, semen, sweat, bile, cerebrospinal fluid, amniotic fluid, and ascites [4, 5].

Released EVs involve in intercellular communication and cellular function regulation under normal physiological conditions, while reprogrammed EVs cargo can lead to an immune response and contribute to the development of diseases under pathological conditions [6]. Various cell types, including natural killer cells, monocytes, dendritic cells, and macrophages [7, 8], have been shown to release EVs to mediate immunostimulatory and immunosuppressive effects by transporting antigens to antigen-presenting cells, activating T cells or inhibiting the activation of regulatory T cells [9]. Accumulating evidence suggested that total EVs, EVs constituents, and EVs surface molecules associate with autoimmune diseases, such as primary Sjögren’s syndrome (pSS), and systemic lupus erythematosus (SLE), oral lichen planus (OLP) [10–14]. Given that, theoretically, EVs can be released by every cell in the body and may increase in pathological conditions [4, 5, 15], EVs have been suggested as promising novel biomarkers [15, 16]. Compared to traditional biomarkers, biological medicines or drug delivery systems, EVs possess several distinct advantages, including (i) capacity to function as noninvasive biomarkers released by almost all cell types and present in almost all body fluids; (ii) ability to reflect the progress of diseases and the effects of treatments through vesicle origin or cargo; (iii) ability to protect natural cargos from freeze/thaw cycles during long-term storage; and (iv) the biodegradability of EVs in body fluids [15, 16].

This review focuses on the EV-associated biomarkers and potential applications of EVs in autoimmune diseases.

### EVs as potential biomarkers in autoimmune diseases

**Fig. 1:**
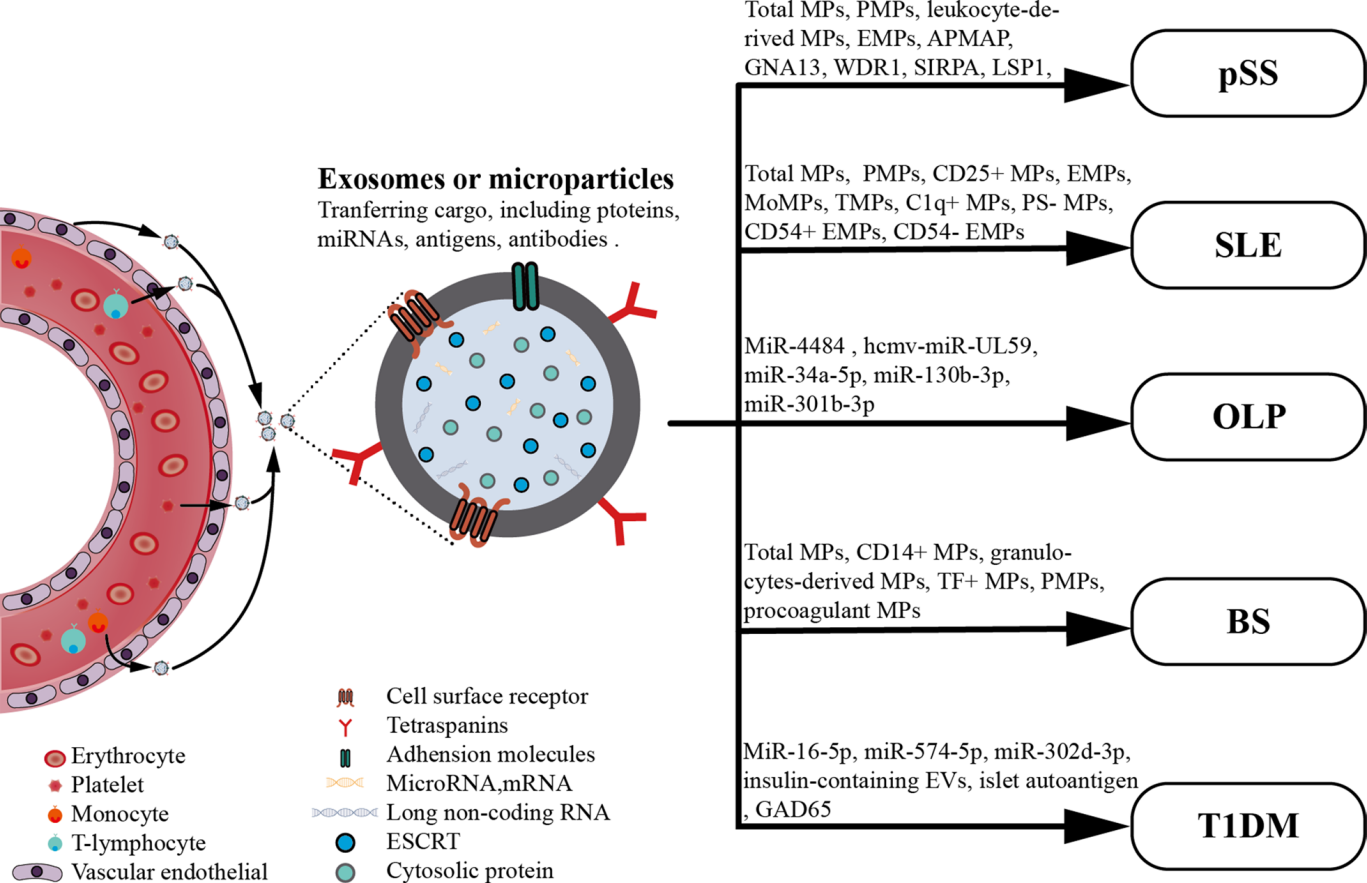
Potential biomarkers in extracellular vesicles (EVs) for autoimmune diseases. *pSS* primary Sjögren’s syndrome, *PMPs* platelet-derived MPs, *EMPs* endothelial MPs, *APMAP* adipocyte plasma membrane-associated protein, *GNA13* guanine nucleotide-binding protein subunit alpha-1, *WDR1* WD repeat-containing protein 1, *SIRPA *tyrosine-protein phosphatase nonreceptor type substrate 1, *LSP1* cell-specific protein 1, *CPNE1* Copine 1, *CALM* Calmodulin, *moMPs* monocyte-derived MPs, *TMPs* T cell-derived MPs, *PS- MPs* phosphatidylserine-negative MPs, *SLE* systemic lupus erythematosus, *OLP* oral lichen planus, *TF + MPs* tissue factor + MPs, *BS* Behçet’s syndrome, *GAD65* glutamic acid decarboxylase 65, *T1DM* type 1 diabetes mellitus

Autoimmune diseases, characterized by self-immune responses, are one of the leading causes of morbidity and mortality among chronic diseases [17]. Imbalance in the activation and regulation of cells can result in dysregulated cell activation, leading to the production of autoantibodies and damage to tissues expressing the target antigen [18]. Considering the increasing number of new cases of autoimmune diseases and the poor understanding of the etiologies of autoimmune diseases that greatly impedes the prevention, diagnosis and treatment of autoimmune diseases, researchers worldwide have been searching for more reliable and convenient biomarkers for autoimmune diseases. Some previous studies have determined that EVs are involved in immunostimulation or immunosuppression in autoimmune diseases through pro-inflammatory or anti-inflammatory effects induced by their specific constituents [10, 14, 15, 19, 20]. Moreover, studies have suggested increasing total EVs levels and specific EVs constituents as potential diagnostic biomarkers in several autoimmune diseases, such as primary Sjögren’s syndrome, systemic lupus erythematosus, and systemic sclerosis [21] (Fig. 1).

### EVs as biomarkers in primary Sjögren’s syndrome

Primary Sjögren’s syndrome, a chronic female‐dominant autoimmune disorder influencing approximately 1% of the general population and 3% of people older than 50 years [21], is characterized by keratoconjunctivitis sicca and xerostomia induced by the focal lymphocytic infiltration in exocrine glands and lacrimal gland.

One previous study reported that although the levels of MPs in pSS patients with high or low disease severity were higher than health controls, those in pSS patients with high disease severity were lower than those in patients with low disease severity(Table 1) [22].The potential explanations include consumption or confinement of MPs by adhesion in the tissue target of pSS, MPs sequestered in leukocyte-platelet complexes, and MPs destruction induced by phospholipases, especially secretory phospholipase A2, in active disease [22–27]. In addition to increased PMPs levels, increased levels of endothelial microparticles (EMPs), which are significantly correlated with the disease duration from symptom onset and diagnosis, were also found in pSS patients compared with healthy controls [28]. Aqrawi et al. identified thirty-six proteins, including adipocyte plasma membrane-associated protein, which correlates with adipocyte differentiation, and SIRPA and LSP1, which are associated with activation of the innate immune system, upregulated in the EVs from saliva of pSS patients compared to controls. They also revealed increased expressions of Copine 1 and Calmodulin in the tears of pSS patients [24]. Study also suggested hsa-mir-768-3p and hsa-mir-574-3p in the minor salivary glands, which are involved in minor salivary gland inflammation and detectable in salivary EVs, to be promising biomarkers in the minor salivary glands reflecting inflammation and salivary gland dysfunction in pSS [29, 30] Overall, these results revealed increased EVs from different biofluids in pSS, as well as changed expressions of specific proteins and miRNAs in EVs. Levels of EVs and specific components of EVs may be promising diagnosis or prognosis markers and reflect the potential underlying mechanisms of pSS.

**Table 1:** EV-associated biomarkers in primary Sjögren’s syndrome

EVs or constituents	Source	Isolation method	Quantification method	References
Total MPs, PMPs, leukocyte-derived MPs	Plasma	Centrifugation	Functional prothrombinase capture assay and flow cytometry	[22]
EMPs	Plasma	Affinity-based capture	Flow cytometry	[28]
APMAP, GNA13, WDR1, SIRPA, LSP1	Saliva	Size-exclusion chromatography	Flow cytometry	[24]
CPNE1, CALM	Tear	Size-exclusion chromatography	Flow cytometry	[24]

*MPs* microparticles, *PMPs* platelet-derived MPs, *EMPs* endothelial MPs, *APMAP* adipocyte plasma membrane-associated protein, *GNA13* guanine nucleotide-binding protein subunit alpha-1, *WDR1* WD repeat-containing protein 1, *SIRPA* tyrosine-protein phosphatase nonreceptor type substrate 1, *LSP1* cell-specific protein 1, *CPNE1* Copine 1, *CALM* Calmodulin

### EVs in systemic lupus erythematosus

Systemic lupus erythematosus, a systemic autoimmune disease influencing multiple organs simultaneously with poor quality of life and substantial mortality, is characterized by the presence of autoreactive T cells and hyperactive B cells that produce autoantibodies forming immune complex deposits [31, 32].

Proven to increase adhesion molecule expressions, chemokine productions, and structural alterations in macrovascular and microvascular endothelial cells, which can lead to endothelial alterations and tissue leukocyte infiltration [10, 33], MPs in the plasma of SLE patients have been intensively studied as elements in “liquid biopsies” for SLE (Table 2).

**Table 2:** EV-associated biomarkers in systemic lupus erythematosus

EVs or cargo in EVs	Source	Isolation method	Quantification method	References
Total MPs	Plasma	Affinity-based capture	Flow cytometry	[35]
Total MPs, PMPs, CD25 + MPs, EMPs, monocyte-derived MPs, and T cell-derived MPs	Plasma	Centrifugation	Flow cytometry	[23]
Total MPs and IgG + MPs	Plasma	Centrifugation	Flow cytometry	[37]
Total MPs, IgM + MPs, and IgG + MPs	Plasma	Centrifugation	Flow cytometry	[38]
IgM + MPs and C1q + MPs	Plasma	Affinity-based capture	Flow cytometry	[11]
CD14 + monocyte-derived MPs	Plasma	Centrifugation	Flow cytometry	[40]
Total MPs and phosphatidylserine-negative MPs	Plasma	Centrifugation	Flow cytometry	[36]
CD41 + MPs harboring IgG and CD41– MP harboring IgG	Plasma	Affinity-based capture	Flow cytometry	[41]
CD31 + /annexin V + /CD42b- EMPs	Plasma	Affinity-based capture	Flow cytometry	[42]
Total EMPs, CD54 + EMPs, CD54- EMPs, and CD54 + EMPs/total EMPs	Plasma	Fluorophore-conjugated mAb staining	Flow cytometry	[34]
Total MPs and PMPs	Plasma	Centrifugation	Flow cytometry and a functional prothrombinase capture assay	[22]

*MPs* microparticles, *PMPs* platelet-derived MPs, *EMPs* endothelial microparticles, *PS- MPs* phosphatidylserine-negative MPs

Many studies have shown increased total MPs levels in the plasma of SLE patients compared with those of healthy controls [22, 34–38]. López et al. proved that total MPs, CD25 + MPs, EMPs, platelet-derived MPs, monocytes or T cells in the plasma of SLE patients associated with the increased disease duration and higher risk of cardiovascular disease [23]. Scientists have also found increased total MPs and IgG + MPs [37, 38], as well as relatively lower IgM + MPs and C1q + MPs in patients with SLE [39]. Another study showed a positive association between plasmatic CD14 + monocyte-derived MPs and disease activity [40]. A subsequent study showed that phosphatidylserine-negative MPs/MPs was increased in SLE patients compared to healthy controls, especially in females and smokers [36]. Moreover, Fortin et al. revealed a positive correlation between CD41 + MPs harboring IgG and the SLE Disease Activity Index 2000, as well as a positive association between the concentrations of CD41– MP harboring IgG and Systemic Lupus International Collaborating Clinics/American College of Rheumatology Damage Index, and carotid US plaques and intima-media thickness [41]. Moreover, a previous study reported higher CD31 + /annexin V + /CD42b- EMPs levels in SLE patients than in healthy controls and an association between CD31 + /annexin V + /CD42b- EMPs and the median global BILAG-2004 score after treatment [42]. Another study found increased EMPs levels and a lower ratio of CD54( +) EMPs/total EMPs in SLE patients, especially in women with moderate-to-high disease activity, compared to controls [34]. In conclusion, EVs mediates intercellular communication between immune cells, endothelial cells, and platelets with the changes of specific components in the development of SLE and provide potential biomarkers for SLE diagnosis and prognosis. These biomarkers may partly implicate the mechanism of SLE and provide new directions for the targeted therapies of SLE.

### EVs in other autoimmune diseases

EV-associated biomarkers have been intensively studied in other autoimmune diseases (Table 3). Oral lichen planus, a T cell-mediated chronic autoimmune disease with a prevalence rate of 0.1–4.0% in the adult population [43, 44], is characterized by keratotic or erythematous lesions in the oral mucosa. The symptoms of OLP could be symmetrical, bilateral, or multiple lesions with different patterns of plaque, reticular, papular, bullous, erosive, and atrophic features [45]. A previous study suggested that different expression patterns of miRNAs in EVs associated with cytokine regulation in OLP patients may contribute to the elucidation of the pathogenesis of OLP [46], and a recent study reported that EVs from the plasma of OLP patients could enhance T cell proliferation and attenuate apoptosis, which might promote the development of OLP [47]. Ding et al. reported increased levels of hcmv-miR-UL59, which is primarily encapsulated in EVs in the plasma, in the plasma of OLP patients [48]. Another study revealed the upregulated expression levels of miR-4484 in salivary EVs from OLP patients and identified this miRNA as a potential biomarker for OLP [45]. In addition, a study reported different expression levels of miR-34a-5p, miR-130b-3p, and miR-301b-3p in circulating EVs in OLP, as well as an association between the level of miR-34a-5p and disease severity [49].

**Table 3:** EV-associated biomarkers in other autoimmune diseases

EVs or cargo in EVs	Source	Isolation method	Quantification method	Biomarkers	References
MiR-4484	Saliva	Precipitation	MiRNA microarray analysis and flow cytometry	OLP	[45]
MiR-34a-5p, miR-130b-3p and miR-301b-3p	Plasma	Membrane affinity -based capture	MiRNA microarray analysis and flow cytometry	OLP	[49]
Hcmv-miR-UL59	Plasma	Precipitation	RT-qPCR analysis	OLP	[48]
Total MPs, CD14 + MPs, Granulocytes-derived MPs, and tissue factor + MPs	Plasma	Affinity-based capture	Flow cytometry	BS	[59]
PMPs	Whole blood	Unreported	Flow cytometry	BS	[60]
Procoagulant MPs	Plasma	Affinity-based capture	Functional prothrombinase capture assay	BS	[61]
MiR-16-5p, miR-574-5p and miR-302d-3p	Plasma	Ultracentrifugation	RT-qPCR analysis	T1DM	[62]
Insulin-containing exosomes, exosomal islet autoantigen and GAD65	Plasma	Size-based filtration	Affinity-based capture and RT-PCR analyses	T1DM	[63]

*OLP* oral lichen planus, *RT-qPCR* Realtime quantitative polymerase chain reaction, *BS* Behçet’s syndrome, *TF* + *MPs* tissue factor + MPs, *PMPs* platelet-derived MPs, *T1DM* type 1 diabetes mellitus, *GAD65* glutamic acid decarboxylase 65

Behçet’s syndrome (BS), a multisystem inflammatory disorder involving venous and arterial vessels [50], is characterized by oral and genital ulceration, mucocutaneous lesions, arthritis, and uveitis [51]. Although the etiopathogenesis of BS is not fully understood, study have suggested an association between BS and activation of the hemostatic system which could be induced by EVs [52]. Studies have shown that clot propagation is affected by tissue factor + MPs, which are also associated with atherosclerosis and venous thromboembolism [53–55], in preclinical models [56–58]. A study reported increased levels of total MPs and tissue factor + MPs in BS patients and a low ratio of TFPI + MPs counts to tissue factor + MPs counts, which associated with clinical thrombosis risk [59]. Furthermore, an increased percentage of platelet-derived MPs and increased procoagulant MPs expressions were found in BS patients [60, 61].

Type 1 diabetes mellitus (T1DM), a disorder caused by an autoimmune response against insulin-producing β cells in the pancreatic islets, is the most severe form of diabetes mellitus. A recent study indicated that EVs play an important role to transfer autoantigen peptides from insulin-producing β cells in the pathogenesis of T1DM [15]. Study had reported upregulated expressions of miRNAs in EVs, including miR-16-5p, miR-574-5p and miR-302d-3p, in the plasma of T1DM patients compared with those of healthy controls [62]. In addition, Korutla reported that insulin-containing EVs from transplanted islets and the cargos in these EVs, including islet autoantigen and glutamic acid decarboxylase 65, could reflect the destruction of transplanted β cells secondary to recurrent T1DM autoimmunity [63]. In summary, further studies are necessary to explore the potential diagnostic and prognostic EVs biomarkers in autoimmune diseases.

### EVs as therapeutic approaches in autoimmune diseases

**Fig. 2:**
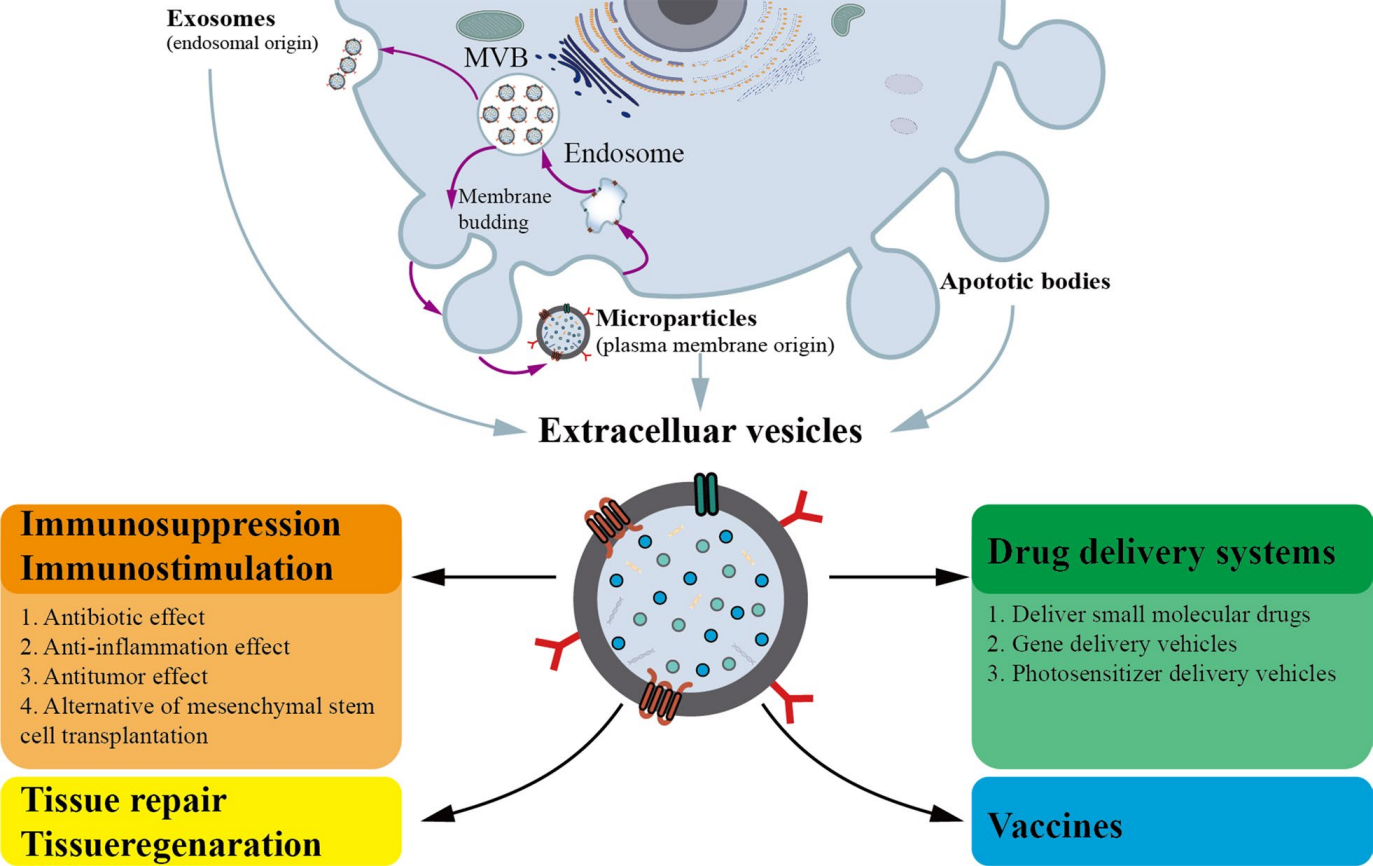
Research aimed at developing extracellular vesicles (EVs) for clinical applications. *MVB* multivesicular body

In addition to the promising use as biomarkers, EVs have been suggested as potential therapeutic approaches which can be divided into four categories: (i) utilizing EVs to transfer the natural cargo of EVs to induce immunosuppressive or immunostimulatory effects, including antimicrobial effects, anti-inflammatory effects, and antitumor effects or utilizing EVs as an alternative to mesenchymal stem cell transplantation; (ii) utilizing bioengineering techniques to modify EVs as nanocarriers for drug delivery systems to deliver specific nucleic acids (miRNAs, siRNAs, and mRNAs), proteins, and therapeutic agents to target cells or tissues; (iii) utilizing EVs to induce tissue regeneration and tissue repair; and (iv) utilizing EVs as novel vaccines in the treatment of tumors or infections (Fig. 2) [20].

## Conclusion

Accumulating evidence supports that EVs involve in intercellular communication inducing immunostimulation and immunosuppression, and EVs are promising biomarkers or therapeutic approaches for autoimmune diseases. In this review, we provided evidence for the biomarker potential of EVs in several autoimmune diseases and summarized the potential use of EVs in therapies. However, both basic and applied studies of EVs are still in the early stages, and the poor understanding of the underlying mechanisms hinders the clinical translation of EVs. Obviously, extensive studies of EVs are necessary before application for the clinical diagnosis, prognosis and therapy of autoimmune diseases can be performed, including (i) studies on the separation and purification of EVs; (ii) studies providing an intensive understanding of EVs biogenesis and targeting; (iii) studies providing an intensive understanding of the mechanism by which EVs induce immunostimulation and immunosuppression; (iv) studies assessing the effect and reliability of EVs as nanodrugs or drug delivery systems in vivo; and (v) studies on clinical applications. Despite the challenges and difficulties remaining before EVs can be clinically applied, their biological and physiological characteristics have shown the great potential of EVs as biomarkers and therapeutic tools. In conclusion, intensive study of the biological functions and mechanisms of EVs could help to identify potential biomarkers and facilitate the clinical translation of EVs.

## Data Availability

The primary data for this study is available from the authors on direct request.

## Abbreviations

EVs: Extracellular vesicles
MPs: Microparticles
pSS: Primary Sjögren’s syndrome
SLE: Systemic lupus erythematosus
OLP: Oral lichen planus
EMPs: Endothelial microparticles
BS: Behçet’s syndrome
T1DM: Type 1 diabetes mellitus

## References

[1] Iwai K, Minamisawa T, Suga K, Yajima Y, Shiba K (2016). Isolation of human salivary extracellular vesicles by iodixanol density gradient ultracentrifugation and their characterizations. J Extracell Vesicles.

[2] Yáñez-Mó M, Siljander PR, Andreu Z, Zavec AB, Borràs FE, Buzas EI, Buzas K, Casal E, Cappello F, Carvalho J (2015). Biological properties of extracellular vesicles and their physiological functions. J Extracell Vesicles.

[3] de Lizarrondo S, Roncal C, Calvayrac O, Rodríguez C, Varo N, Purroy A, Lorente L, Rodríguez JA, Doeuvre L, Hervás-Stubbs S (2012). Synergistic effect of thrombin and CD40 ligand on endothelial matrix metalloproteinase-10 expression and microparticle generation in vitro and in vivo. Arterioscler Thromb Vasc Biol.

[4] Fuster-Matanzo A, Gessler F, Leonardi T, Iraci N, Pluchino S (2015). Acellular approaches for regenerative medicine: on the verge of clinical trials with extracellular membrane vesicles?. Stem Cell Res Ther.

[5] Kalra H, Drummen GP, Mathivanan S (2016). Focus on extracellular vesicles: introducing the next small big thing. Int J Mol Sci.

[6] Raposo G, Stoorvogel W (2013). Extracellular vesicles: exosomes, microvesicles, and friends. J Cell Biol.

[7] Ismail N, Wang Y, Dakhlallah D, Moldovan L, Agarwal K, Batte K, Shah P, Wisler J, Eubank TD, Tridandapani S (2013). Macrophage microvesicles induce macrophage differentiation and miR-223 transfer. Blood.

[8] Lugini L, Cecchetti S, Huber V, Luciani F, Macchia G, Spadaro F, Paris L, Abalsamo L, Colone M, Molinari A (2012). Immune surveillance properties of human NK cell-derived exosomes. J Immunol.

[9] Martin RK, Brooks KB, Henningsson F, Heyman B, Conrad DH (2014). Antigen transfer from exosomes to dendritic cells as an explanation for the immune enhancement seen by IgE immune complexes. PLoS ONE.

[10] Lam KCK, Lam MKN, Chim CS, Chan GCF, Li JCB: The functional role of surface molecules on extracellular vesicles in cancer, autoimmune diseases, and coagulopathy. *J Leukoc Biol* 2020. 10.1002/JLB.3MR0420-067R 32480430

[11] Nielsen CT, Østergaard O, Stener L, Iversen LV, Truedsson L, Gullstrand B, Jacobsen S, Heegaard NH (2012). Increased IgG on cell-derived plasma microparticles in systemic lupus erythematosus is associated with autoantibodies and complement activation. Arthritis Rheum.

[12] Zhang HG, Liu C, Su K, Yu S, Zhang L, Zhang S, Wang J, Cao X, Grizzle W, Kimberly RP (2006). A membrane form of TNF-alpha presented by exosomes delays T cell activation-induced cell death. J Immunol.

[13] Robbins PD, Morelli AE (2014). Regulation of immune responses by extracellular vesicles. Nat Rev Immunol.

[14] Turpin D, Truchetet ME, Faustin B, Augusto JF, Contin-Bordes C, Brisson A, Blanco P, Duffau P (2016). Role of extracellular vesicles in autoimmune diseases. Autoimmun Rev.

[15] Tian J, Casella G, Zhang Y, Rostami A, Li X (2020). Potential roles of extracellular vesicles in the pathophysiology, diagnosis, and treatment of autoimmune diseases. Int J Biol Sci.

[16] Lai RC, Yeo RW, Tan KH, Lim SK (2013). Exosomes for drug delivery - a novel application for the mesenchymal stem cell. Biotechnol Adv.

[17] Lerner A, Patricia W, Matthias T (2015). The world incidence and prevalence of autoimmune diseases is increasing. Int J Celiac Dis.

[18] Rosenblum MD, Remedios KA, Abbas AK (2015). Mechanisms of human autoimmunity. J Clin Invest.

[19] Tan L, Wu H, Liu Y, Zhao M, Li D, Lu Q (2016). Recent advances of exosomes in immune modulation and autoimmune diseases. Autoimmunity.

[20] Katsiougiannis S (2015). Extracellular vesicles: evolving contributors in autoimmunity. For Immunopathol Dis Therap.

[21] Cecchettini A, Finamore F, Puxeddu I, Ferro F, Baldini C (2019). Salivary extracellular vesicles versus whole saliva: new perspectives for the identification of proteomic biomarkers in Sjögren's syndrome. Clin Exp Rheumatol.

[22] Sellam J, Proulle V, Jüngel A, Ittah M, Miceli Richard C, Gottenberg JE, Toti F, Benessiano J, Gay S, Freyssinet JM, Mariette X (2009). Increased levels of circulating microparticles in primary Sjögren's syndrome, systemic lupus erythematosus and rheumatoid arthritis and relation with disease activity. Arthritis Res Ther.

[23] López P, Rodríguez-Carrio J, Martínez-Zapico A, Caminal-Montero L, Suárez A (2017). Circulating microparticle subpopulations in systemic lupus erythematosus are affected by disease activity. Int J Cardiol.

[24] Aqrawi LA, Galtung HK, Vestad B, Øvstebø R, Thiede B, Rusthen S, Young A, Guerreiro EM, Utheim TP, Chen X (2017). Identification of potential saliva and tear biomarkers in primary Sjögren's syndrome, utilising the extraction of extracellular vesicles and proteomics analysis. Arthritis Res Ther.

[25] Dieker J, Tel J, Pieterse E, Thielen A, Rother N, Bakker M, Fransen J, Dijkman HB, Berden JH, de Vries JM (2016). Circulating apoptotic microparticles in systemic lupus erythematosus patients drive the activation of dendritic cell subsets and prime neutrophils for NETosis. Arthritis Rheumatol.

[26] Crookston KP, Sibbitt WL, Chandler WL, Qualls CR, Roldan CA (2013). Circulating microparticles in neuropsychiatric systemic lupus erythematosus. Int J Rheum Dis.

[27] Nielsen CT, Østergaard O, Johnsen C, Jacobsen S, Heegaard NH (2011). Distinct features of circulating microparticles and their relationship to clinical manifestations in systemic lupus erythematosus. Arthritis Rheum.

[28] Bartoloni E, Alunno A, Bistoni O, Caterbi S, Luccioli F, Santoboni G, Mirabelli G, Cannarile F, Gerli R (2015). Characterization of circulating endothelial microparticles and endothelial progenitor cells in primary Sjögren's syndrome: new markers of chronic endothelial damage?. Rheumatology (Oxford).

[29] Michael A, Bajracharya SD, Yuen PS, Zhou H, Star RA, Illei GG, Alevizos I (2010). Exosomes from human saliva as a source of microRNA biomarkers. Oral Dis.

[30] Alevizos I, Alexander S, Turner RJ, Illei GG (2011). MicroRNA expression profiles as biomarkers of minor salivary gland inflammation and dysfunction in Sjögren's syndrome. Arthritis Rheum.

[31] Al-Shobaili HA, Al Robaee AA, Alzolibani AA, Rasheed Z (2012). Antibodies against 4-hydroxy-2-nonenal modified epitopes recognized chromatin and its oxidized forms: role of chromatin, oxidized forms of chromatin and 4-hydroxy-2-nonenal modified epitopes in the etiopathogenesis of SLE. Dis Markers.

[32] Colasanti T, Maselli A, Conti F, Sanchez M, Alessandri C, Barbati C, Vacirca D, Tinari A, Chiarotti F, Giovannetti A (2012). Autoantibodies to estrogen receptor α interfere with T lymphocyte homeostasis and are associated with disease activity in systemic lupus erythematosus. Arthritis Rheum.

[33] Atehortúa L, Rojas M, Vásquez G, Muñoz-Vahos CH, Vanegas-García A, Posada-Duque RA, Castaño D (2019). Endothelial activation and injury by microparticles in patients with systemic lupus erythematosus and rheumatoid arthritis. Arthritis Res Ther.

[34] Duval A, Helley D, Capron L, Youinou P, Renaudineau Y, Dubucquoi S, Fischer AM, Hachulla E (2010). Endothelial dysfunction in systemic lupus patients with low disease activity: evaluation by quantification and characterization of circulating endothelial microparticles, role of anti-endothelial cell antibodies. Rheumatology (Oxford).

[35] Pereira J, Alfaro G, Goycoolea M, Quiroga T, Ocqueteau M, Massardo L, Pérez C, Sáez C, Panes O, Matus V, Mezzano D (2006). Circulating platelet-derived microparticles in systemic lupus erythematosus. Association with increased thrombin generation and procoagulant state. Thromb Haemost.

[36] Mobarrez F, Vikerfors A, Gustafsson JT, Gunnarsson I, Zickert A, Larsson A, Pisetsky DS, Wallén H, Svenungsson E (2016). Microparticles in the blood of patients with systemic lupus erythematosus (SLE): phenotypic characterization and clinical associations. Sci Rep.

[37] Ullal AJ, Reich CF, Clowse M, Criscione-Schreiber LG, Tochacek M, Monestier M, Pisetsky DS (2011). Microparticles as antigenic targets of antibodies to DNA and nucleosomes in systemic lupus erythematosus. J Autoimmun.

[38] Burbano C, Villar-Vesga J, Orejuela J, Muñoz C, Vanegas A, Vásquez G, Rojas M, Castaño D (2018). potential involvement of platelet-derived microparticles and microparticles forming immune complexes during monocyte activation in patients with systemic lupus erythematosus. Front Immunol.

[39] Østergaard O, Nielsen CT, Iversen LV, Tanassi JT, Knudsen S, Jacobsen S, Heegaard NH (2013). Unique protein signature of circulating microparticles in systemic lupus erythematosus. Arthritis Rheum.

[40] Viñuela-Berni V, Doníz-Padilla L, Figueroa-Vega N, Portillo-Salazar H, Abud-Mendoza C, Baranda L, González-Amaro R (2015). Proportions of several types of plasma and urine microparticles are increased in patients with rheumatoid arthritis with active disease. Clin Exp Immunol.

[41] Fortin PR, Cloutier N, Bissonnette V, Aghdassi E, Eder L, Simonyan D, Laflamme N, Boilard E (2016). Distinct subtypes of microparticle-containing immune complexes are associated with disease activity, damage, and carotid intima-media thickness in systemic lupus erythematosus. J Rheumatol.

[42] Parker B, Al-Husain A, Pemberton P, Yates AP, Ho P, Gorodkin R, Teh LS, Alexander MY, Bruce IN (2014). Suppression of inflammation reduces endothelial microparticles in active systemic lupus erythematosus. Ann Rheum Dis.

[43] Arão TC, Guimarães AL, de Paula AM, Gomes CC, Gomez RS (2012). Increased miRNA-146a and miRNA-155 expressions in oral lichen planus. Arch Dermatol Res.

[44] Danielsson K, Ebrahimi M, Wahlin YB, Nylander K, Boldrup L (2012). Increased levels of COX-2 in oral lichen planus supports an autoimmune cause of the disease. J Eur Acad Dermatol Venereol.

[45] Byun JS, Hong SH, Choi JK, Jung JK, Lee HJ (2015). Diagnostic profiling of salivary exosomal microRNAs in oral lichen planus patients. Oral Dis.

[46] Ma H, Wu Y, Yang H, Liu J, Dan H, Zeng X, Zhou Y, Jiang L, Chen Q (2016). MicroRNAs in oral lichen planus and potential miRNA-mRNA pathogenesis with essential cytokines: a review. Oral Surg Oral Med Oral Pathol Oral Radiol.

[47] Peng Q, Zhang J, Zhou G (2019). Circulating exosomes regulate T-cell-mediated inflammatory response in oral lichen planus. J Oral Pathol Med.

[48] Ding M, Wang X, Wang C, Liu X, Zen K, Wang W, Zhang CY, Zhang C (2017). Distinct expression profile of HCMV encoded miRNAs in plasma from oral lichen planus patients. J Transl Med.

[49] Peng Q, Zhang J, Zhou G (2018). Differentially circulating exosomal microRNAs expression profiling in oral lichen planus. Am J Transl Res.

[50] Jennette JC, Falk RJ, Bacon P, Basu N, Cid Xutgla M, Ferrario F, Flores-Suarez LF, Gross W, Guillevin L, Hagen EC (2013). Revised international chapel hill consensus conference nomenclature of vasculitides. Arthritis Rheum.

[51] Ambrose NL, Haskard DO (2013). Differential diagnosis and management of Behçet syndrome. Nat Rev Rheumatol.

[52] Lacroix R, Dubois C, Leroyer AS, Sabatier F, Dignat-George F (2013). Revisited role of microparticles in arterial and venous thrombosis. J Thromb Haemost.

[53] Ye R, Ye C, Huang Y, Liu L, Wang S (2012). Circulating tissue factor positive microparticles in patients with acute recurrent deep venous thrombosis. Thromb Res.

[54] Morel O, Pereira B, Averous G, Faure A, Jesel L, Germain P, Grunebaum L, Ohlmann P, Freyssinet JM, Bareiss P, Toti F (2009). Increased levels of procoagulant tissue factor-bearing microparticles within the occluded coronary artery of patients with ST-segment elevation myocardial infarction: role of endothelial damage and leukocyte activation. Atherosclerosis.

[55] Hron G, Kollars M, Weber H, Sagaster V, Quehenberger P, Eichinger S, Kyrle PA, Weltermann A (2007). Tissue factor-positive microparticles: cellular origin and association with coagulation activation in patients with colorectal cancer. Thromb Haemost.

[56] Engelmann B, Massberg S (2013). Thrombosis as an intravascular effector of innate immunity. Nat Rev Immunol.

[57] Reinhardt C, von Brühl ML, Manukyan D, Grahl L, Lorenz M, Altmann B, Dlugai S, Hess S, Konrad I, Orschiedt L (2008). Protein disulfide isomerase acts as an injury response signal that enhances fibrin generation via tissue factor activation. J Clin Invest.

[58] Chou J, Mackman N, Merrill-Skoloff G, Pedersen B, Furie BC, Furie B (2004). Hematopoietic cell-derived microparticle tissue factor contributes to fibrin formation during thrombus propagation. Blood.

[59] Khan E, Ambrose NL, Ahnström J, Kiprianos AP, Stanford MR, Eleftheriou D, Brogan PA, Mason JC, Johns M, Laffan MA, Haskard DO (2016). A low balance between microparticles expressing tissue factor pathway inhibitor and tissue factor is associated with thrombosis in Behçet's Syndrome. Sci Rep.

[60] Macey M, Hagi-Pavli E, Stewart J, Wallace GR, Stanford M, Shirlaw P, Fortune F (2011). Age, gender and disease-related platelet and neutrophil activation ex vivo in whole blood samples from patients with Behçet's disease. Rheumatology (Oxford).

[61] Mejía JC, Ortiz T, Tàssies D, Solanich X, Vidaller A, Cervera R, Reverter JC, Espinosa G (2016). Procoagulant microparticles are increased in patients with Behçet's disease but do not define a specific subset of clinical manifestations. Clin Rheumatol.

[62] Garcia-Contreras M, Shah SH, Tamayo A, Robbins PD, Golberg RB, Mendez AJ, Ricordi C (2017). Plasma-derived exosome characterization reveals a distinct microRNA signature in long duration Type 1 diabetes. Sci Rep.

[63] Korutla L, Rickels MR, Hu RW, Freas A, Reddy S, Habertheuer A, Harmon J, Korutla V, Ram C, Naji A, Vallabhajosyula P (2019). Noninvasive diagnosis of recurrent autoimmune type 1 diabetes after islet cell transplantation. Am J Transplant.

